# High-Sensitivity, Large-Doppler-Range DSSS–GMSK Signal Acquisition Algorithm for Earth–Moon Space Satellite Communication

**DOI:** 10.3390/s26165191

**Published:** 2026-08-17

**Authors:** Yuanshen Lu, Min Wu, Wen Zhang, Yu Zhu, Qihao Xia, Jun Zhang

**Affiliations:** 1 Innovation Research Institute of Microsatellite of Chinese Academy of Sciences, Shanghai 201304, China; zw@microsate.com (W.Z.); zhuyu@micosate.com (Y.Z.); xiaqh@micosate.com (Q.X.); zhangjun@microsate.com (J.Z.); 2 Shanghai Engineering Center for Microsatellites, Shanghai 201203, China

**Keywords:** direct-sequence spread spectrum, Gaussian-filtered minimum-shift keying, lunar and deep-space exploration, TDRSS, wide-Doppler-range signal acquisition, high-sensitivity signal acquisition

## Abstract

With the intensifying competition among countries in lunar and deep-space exploration, the forward communication bands between Earth and the Moon are becoming increasingly congested. Spread-spectrum communication is poised to replace conventional unified-carrier wave systems as the dominant telemetry and tracking technology. Compared with Phase-Shift Keying (PSK) modulation, Gaussian-Filtered Minimum-Shift Keying (GMSK) offers lower transmit sidelobes and higher spectral efficiency, making it a promising alternative to Quadrature Phase-Shift Keying (QPSK) as a communication scheme integrated with spread-spectrum systems. This paper compares the direct-sequence spread-spectrum Gaussian-Filtered Minimum-Shift Keying (DSSS–GMSK) modulation scheme with the widely adopted DSSS–BPSK scheme and demonstrates the advantages of DSSS–GMSK, including higher spectral efficiency and improved signal acquisition performance over a wide Doppler dynamic range. This paper also proposes a deep-space DSSS–GMSK signal acquisition algorithm capable of achieving signal acquisition at a carrier-to-noise density ratio $\left(C/N_{0}\right)$ of 34 dB-Hz over a Doppler range from −350 kHz to +350 kHz, demonstrating the feasibility and potential of DSSS–GMSK. The algorithm requires minimal computational hardware resources, and its feasibility has been verified through simulations. Furthermore, its resistance to false locking is analyzed and discussed.

## 1. Introduction

In recent years, countries have shown a growing interest in cislunar exploration initiatives. In 2019, the United States released the Next Generation Defense Space Architecture and launched the ‘Artemis’ program [1], which notably includes a plan to deploy the ‘Gateway’ cislunar relay communication hub satellite into a Near-Rectilinear Halo Orbit (NRHO) by 2030. Meanwhile, the European Space Agency (ESA) has initiated the “Moonlight” program to validate near-lunar-orbit satellites and lunar surface static ranging beacon stations.

China has also undertaken several cislunar projects in recent years. These include the 2018 launch of the “Queqiao” relay satellite employing X-band reverse link communications [2], as well as leading and participating in the International Lunar Research Station (ILRS) initiative [3], which is positioned as a counterpart to the Artemis program.

The National Aeronautics and Space Administration (NASA) has published guidelines for communications within cislunar space. For communications with the Gateway satellite, suggested modulation schemes include filtered Offset Quadrature Phase-Shift Keying (filtered-OQPSK) and Gaussian Minimum-Shift Keying (GMSK) [4]. These recommendations aim to reduce frequency interference and improve spectral efficiency. Future forward link data rates from Earth to the Moon are projected to reach hundreds of megabits per second. As demand for cislunar communication frequency resources intensifies, future developments may include not only the adoption of higher-frequency Q/V bands but also the use of code-division multiplexing in the X-band or Ka-band to enable extensive frequency reuse [5].

In 2024, the Chinese Academy of Sciences launched a cislunar space exploration satellite into a distant retrograde orbit (DRO). This satellite is equipped with the first deep-space X-band spread-spectrum communication transponder. The transponder supports high-sensitivity acquisition of spread-spectrum BPSK signals and enables search capabilities across a Doppler frequency range of ±350 kHz while accommodating Doppler shift rates up to the order of 5 kHz/s. It has been successfully launched and has undergone in-orbit technical verification.

The main objective of this study is to highlight the advantages of GMSK-based spread-spectrum systems in deep-space communication scenarios and to propose a GMSK-based spread-spectrum signal acquisition algorithm that effectively handles large Doppler shifts, achieves high acquisition sensitivity, and is readily implemented on spacecraft communication processors, thereby demonstrating its practical applicability.

The organization of this paper is as follows. Section 2 presents the DSSS–GMSK modulation scheme and recommends its application for satellite Telemetry, Tracking, and Control (TT&C), and highlights the advantages of DSSS–GMSK over the widely employed DSSS–BPSK in practical satellite TT&C communications. Section 3 analyzes the DSSS–GMSK signal acquisition methods. Several subsections in Section 3 discuss the integration duration, matched filter length, and estimated acquisition time, then introduce an acquisition approach that balances efficiency and resource utilization. Section 4 presents simulation results on the performance of the acquisition approach and discusses the robustness of the proposed algorithm against false locks caused by the same-frequency, different-code groups. Section 5 is the conclusion.

## 2. Introduction of Direct-Sequence Spread-Spectrum (DSSS) Combined with Gaussian Minimum-Shift Keying (GMSK)

### 2.1. A DSSS–GMSK Modulation Scheme for Deep-Space Telemetry, Tracking, and Command (TT&C)

Reference [6] applies GMSK to frequency-hopping communication, primarily aiming to achieve an integrated covert anti-jamming communication and ranging system. Its main application scenario is military communications. In the work of Reference [7], the ranging code and data signal are simultaneously modulated onto the carrier phase using a scheme similar to unified carrier modulation. This approach introduces additional subcarriers into the spectrum, resulting in spectrum broadening, which is unfavorable for alleviating congestion in the X-band (TT&C) frequency allocations mentioned previously. Reference [8] proposes applying direct-sequence spread spectrum (DSSS) to GMSK signals. The main objective is to improve the bit error rate (BER) performance of GMSK under non-coherent demodulation, approaching that of BPSK. However, this work is not intended for deep-space (TT&C) communications. Reference [9] utilizes a locally generated GMSK signal modulated with a PN code to perform despreading prior to demodulation of the spread-spectrum GMSK signal, thereby improving performance at relatively low signal-to-noise ratios. Nevertheless, the SNR considered in that study is still significantly higher than that typically encountered in deep space communications, and no Doppler effects were taken into account.

**Figure 1:**
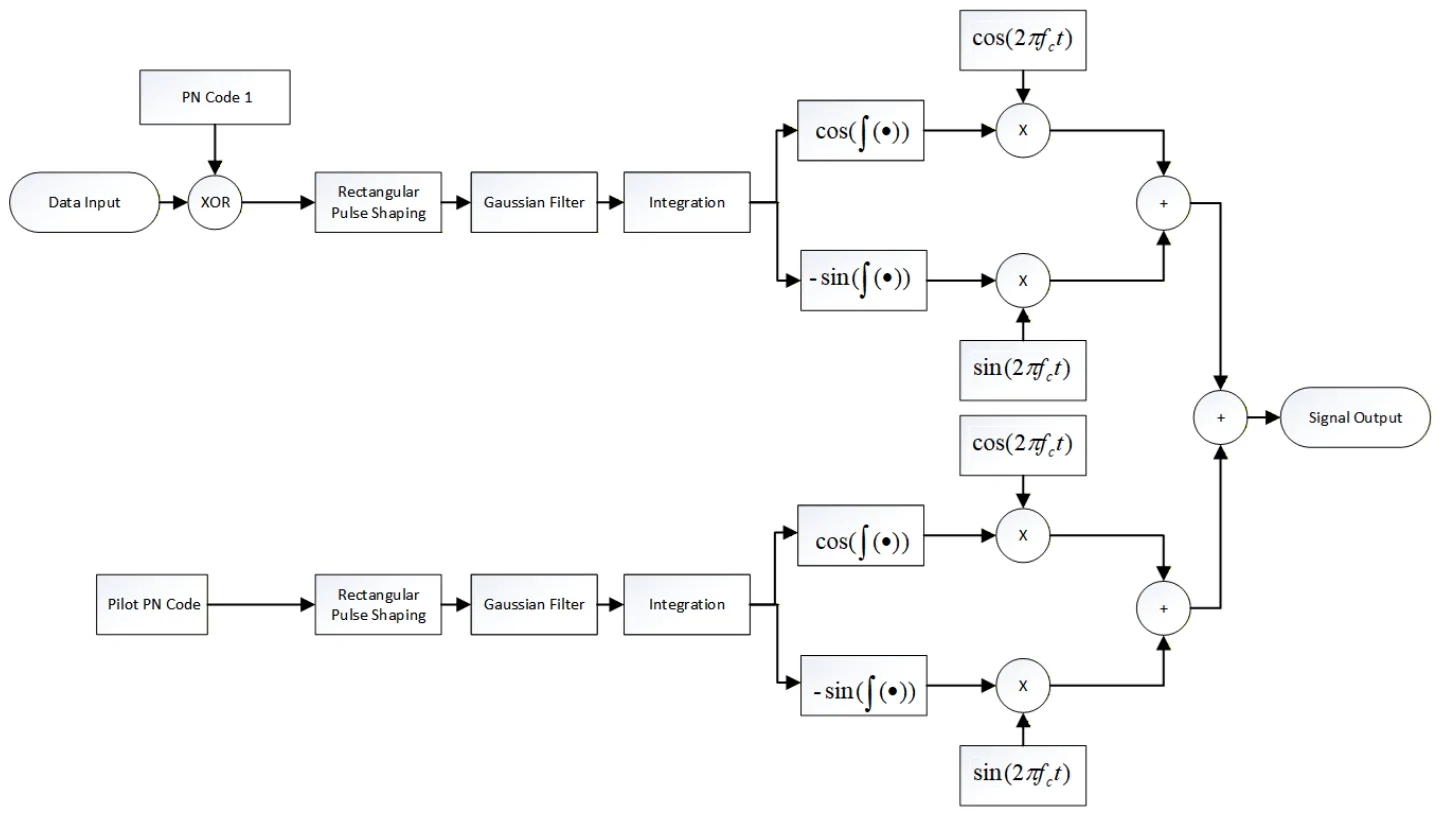
Modulation of the DSSS-GMSK signal for Deep-Space Telemetry, Tracking, and Command.

The DSSS–GMSK phase modulation scheme for Deep-Space Telemetry, Tracking, and Command (TT&C) is illustrated in Figure 1.

A sequence of 1023 PN codes of length is indicated by $c_{n}^{1}$, i = 0, 1, …1022, 0, 1, …, $c_{n}^{1}\in \left[-1,1\right]$. Let the chip interval be $T_{c}$. This PN sequence is multiplied by the bipolar data stream $D_{i}$, generating a new bipolar bit stream denoted by $b_{i}$. The bit stream is first shaped by a rectangular pulse and then passed through a Gaussian filter to form the baseband signal. The resulting baseband signal is integrated to obtain the modulation phase $G_{1}\left(t\right)$. The $S_{data}\left(t\right)$ and $G_{1}\left(t\right)$ expression can be expressed as follows [10]:

(1) $$\begin{array}{c} G_{1}\left(t\right)=\frac{\pi }{2}\sum_{n=0}^{N-1}Gauss\left(b_{n}rect\left(\frac{t-nT_{c}}{T_{c}}\right)\right) \end{array}$$

(2) $$\begin{array}{c} S_{data}\left(t\right)=cos\left(2\pi f_{c}t+G_{1}\left(t\right)\right) \end{array}$$

A pilot-assisted code acquisition is considered, where the transmitted signal consists of two components: a pilot sequence and a data-modulated sequence. Both sequences are phase-aligned and utilize two sets of pseudorandom (PN) codes with the same period. The pilot sequence is modulated solely by the spreading code $c_{n}^{0}$ without any data modulation. Code acquisition is performed on the pilot sequence to estimate the code phase and Doppler information of the data-modulated sequence.

The use of a pilot branch in acquisition is intended to mitigate the impact of data bit flip during long-term integration. Subsequent sections of this paper also discuss the effect of identical-frequency but different-code groups on acquisition performance. Alternatively, instead of employing the pilot branch, one may adopt the Half-Bits Method [10] or the Full-Bits Method [11] to counteract data bit flip. However, these approaches are beyond the scope of this work.

(3) $$\begin{array}{c} S_{Pilot}\left(t\right)=cos\left(2\pi f_{c}t+G_{0}\left(t\right)\right) \end{array}$$

(4) $$\begin{array}{c} G_{0}\left(t\right)=\frac{\pi }{2}\sum_{n=0}^{N-1}Gauss\left(c_{n}^{0}rect\left(\frac{t-nT_{c}}{T_{c}}\right)\right) \end{array}$$

(5) $$\begin{array}{c} Signal\left(t\right)=S_{Pilot}\left(t\right)+S_{data}\left(t\right). \end{array}$$

### 2.2. Comparison of DSSS–GMSK with DSSS–BPSK

Compared with the currently predominant Direct-Sequence Spread-Spectrum Binary Phase-Shift Keying (DSSS–BPSK) modulation used in satellite Telemetry, Tracking, and Control (TT&C), the Direct-Sequence Spread-Spectrum Gaussian Minimum-Shift Keying (DSSS–GMSK) scheme offers three advantages.

**Figure 2:**
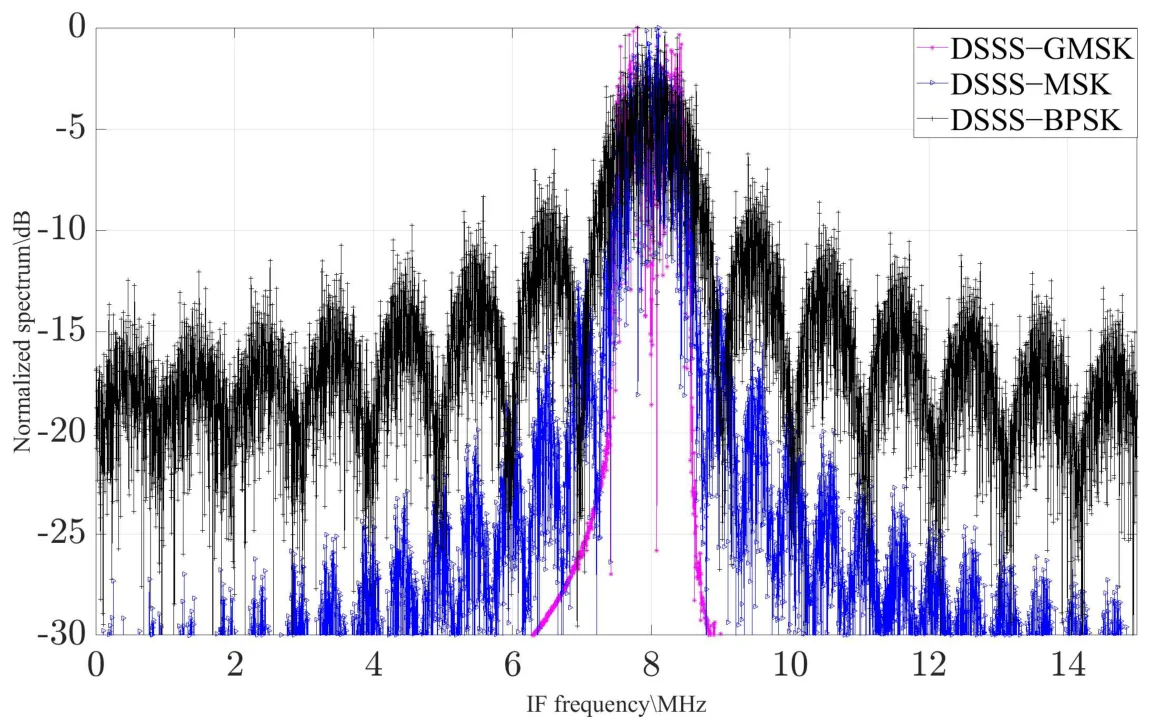
Spectrum of DSSS–GMSK, DSSS–QPSK, and DSSS–BPSK.

1. Because of its phase continuity and rapid out-of-band attenuation, GMSK provides superior spectral efficiency, as shown in Figure 2 [12];
2. GMSK exhibits lower envelope fluctuation, resulting in less performance degradation when transmitted through non-linear channels, which also allows operation in amplifier regions with lower linearity [13];
3. GMSK modulation produces separate in-phase (I) and quadrature (Q) components [14], each with half the total code rate. This feature reduces the impact of Doppler uncertainty on chip misalignment and allows for a simplified acquisition architecture with fewer parallel correlators.

To illustrate 1, Figure 2 presents the normalized spectra of DSSS–GMSK, DSSS–MSK, and DSSS–BPSK. The center frequency is set at an intermediate frequency of 8 MHz, and the spreading code rate is 1.023 MHz. The Gaussian filter shaping factor is set to 0.3. It can be observed that DSSS–BPSK exhibits significant sidelobes, with an attenuation of approximately 10 dBc at ±1.5 MHz. DSSS–MSK, which corresponds to DSSS–GMSK without shaping filtering, also exhibits sidelobes. However, sidelobe suppression is considerably better, showing an attenuation of 17 dBc at ±1.5 MHz. In contrast, DSSS–GMSK with shaping filtering does not exhibit prominent sidelobes, achieving an attenuation of 30 dBc at ±1.5 MHz.

With respect to the second point, ideal BPSK and QPSK waveforms exhibit an instantaneous 0°–180° phase transition at symbol boundaries and are thus regarded as constant-envelope signals. In practice, pulse-shaping filtering smooths the symbol transitions, causing the phase to evolve gradually rather than abruptly. As a result, BPSK and QPSK no longer maintain a strictly constant-envelope property in realistic transmission scenarios [15].

In comparison, the phase of GMSK is generated through a continuous integration of the Gaussian-filtered baseband signal, ensuring smooth phase trajectories even after pulse shaping. This yields an approximately constant envelope, allowing the use of highly efficient Class-C power amplifiers and consequently reducing transmitter cost [16].

To illustrate 3, the chip misalignment arises from the Doppler effect, which causes a mismatch between the chip duration transmitted from the ground and the chip duration actually received on board. When long-term integration of the signal is performed with a fixed sampling interval during acquisition, the chip drift effect may render the integration invalid.

**Figure 3:**
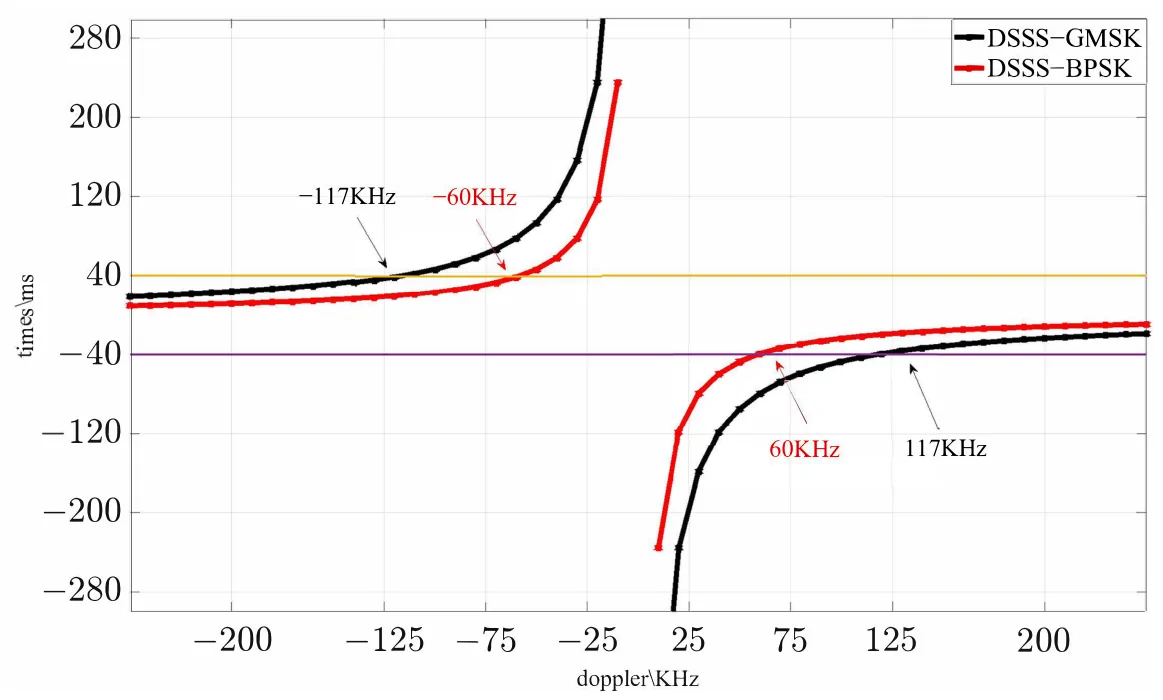
Impact of the Doppler effect on the code chip drift.

Assume that the spreading chip rate is $R_{c}$. For DSSS–GMSK, the in-phase (I) and quadrature (Q) branches have chip rates of $R_{c}/2$, respectively. Considering an X-band center frequency of 7200 MHz and $R_{c}$ = $3.069\times 10^{6}$ chips/s, Figure 3 illustrates the relationship between the Doppler frequency offset (caused by the Doppler effect between the satellite and the ground station) and the time at which the first chip drift occurs. The horizontal axis represents the Doppler frequency offset, while the vertical axis denotes the time of the first chip drift. The black lines correspond to DSSS–GMSK, whereas the red lines correspond to DSSS–BPSK. It can be observed that if the Doppler frequency offset is less than ±117 kHz, no chip drift occurs within 40 ms for DSSS–GMSK. Similarly, if the Doppler frequency offset is less than ±60 kHz, no chip drift occurs within 40 ms for DSSS–BPSK.

## 3. Analysis of DSSS–GMSK Signal Acquisition

### 3.1. Uplink Budget Calculation for a Distant Retrograde Orbit (DRO)

The DRO satellite undergoes a translunar transfer trajectory. After several months of orbit adjustments, it finally reaches the nominal Distant Retrograde Orbit (DRO). During the transfer phase, the maximum distance from Earth may reach 1,500,000 km.

Considering a 15 m aperture ground station antenna from the Chinese Academy of Sciences Institute, and a transmission power of 100 W. The uplink budget is calculated for the apogee of the DRO trajectory. Taking into account various losses, the minimum signal power (that is, the power received at the transponder input) is determined to be −138 dBm, as calculated in Table 1.

### 3.2. Integration Time Analysis

As previously analyzed, during the Earth–Moon DRO insertion phase, the carrier-to-noise power spectral density ratio $\left(C/N_{0}\right)$ is approximately 34 dB-Hz. Given a spread spectrum code rate of $3.069\times 10^{6}$ chips/s, the energy per chip to noise density ratio is as follows:

(6) $$\begin{array}{c} E_{Rc}/N_{0}=34-10*log10\left(3069000\right)=-30.87\;\mathrm{dB-Hz}\; \end{array}$$

Assuming an ideal correlation over a short code sequence of length 1023, and denoting the number of code periods used for integration as *N* we model the noise as additive white Gaussian noise (AWGN). After integrating over *N* code periods, the standard deviation of the accumulated noise amplitude is $0.5(10log10\left(1023N\right)-E_{Rc}/N_{0})$, while the signal benefits from a coherent integration gain of 10log10(1023N) [10]. To ensure a 99% confidence margin where the peak correlation value exceeds all others by at least twice the noise amplitude margin, we apply a 2.58σ criterion. Under $\left(C/N_{0}\right)$ of 34 dB-Hz, this corresponds to a required integration time of approximately 10 ms, which implies N=30, which is calculated by Equation (7):

(7) $$\begin{array}{c} 10log_{10}\left(1023N\right)-10log_{10}\left(2.58\right)=0.5\left(10log_{10}\left(1023N\right)-E_{Rc}/N_{0}\right)+3 \end{array}$$

Typically, to acquire the spread-spectrum signal, a two-dimensional search must be performed on the code phase and Doppler shift. For the Doppler search, the range is from –350 to +350 kHz with a step size of 1 kHz and 1023 code phase positions. The total time of calculation is 700 × 1023 = 716,100. Assuming a 10 ms integration time per calculation, the search would require 7160 s for a single correlator. Even with ten parallel channels, the full-spectrum search would still take 716 s. To address this, a parallel-matched filter with coherent FFT accumulation technique (PMF–FFT) is adopted [17,18]. The PMF–FFT approach effectively transforms the two-dimensional Doppler–code phase search into several independent one-dimensional code phase searches, significantly reducing acquisition time and computational burden.

In practice, various factors contribute to coherent integration losses. These include Doppler-induced phase shifts, filter implementation losses, and partial misalignment of the incoming code sequence. These combined effects typically result in an estimated degradation of 5–6 dB. Therefore, a practical choice for the coherent integration duration per acquisition attempt is 40 ms.

### 3.3. Analysis of Parallel Matched Filter

By appropriately partitioning the two-dimensional search grid of code phase and Doppler frequency, and employing parallel PMF–FFT coherent accumulation, the two-dimensional search is transformed into several independent one-dimensional searches. Upon completion of the full grid search, a global peak selection is performed, followed by threshold detection based on the peak-to-average ratio and primary-to-secondary peak ratio. The acquisition of the signal is achieved through multiple rounds of code phase, Doppler correlation, and detection [15].

According to the PMF–FFT gain expression [12], where L is the number of partial correlation points; K is the number of correlators; $f_{d}$ is the Doppler frequency offset; and N is the number of points input to the FFT module. $\theta_{1}=\pi f_{d}T_{c}$ and $\theta_{2}=\pi Lf_{d}T_{c}-k\pi /N$ in Equation (8). The gain expression contains two terms. The first term is solely dependent on $f_{d}$, which reaches its maximum when $f_{d}$→0. The second term reaches its maximum when $k=f_{d}LNT_{c}$. However, due to the randomness of $f_{d}$, the condition $k=f_{d}LNT_{c}$ is often not satisfied with the integer *k* resulting in scalloping loss. In general, $\theta_{1}<<1$ is required; otherwise, the acquisition gain will be significantly reduced.

(8) $$\begin{array}{ccc} G(f_{d},k) & = & \frac{1}{LK}\frac{\sin(L\theta_{1})}{\sin(\theta_{1})}\frac{\sin(K\theta_{2})}{\sin(\theta_{2})}e^{i\varphi (f_{d},k)} \end{array}$$

**Figure 4:**
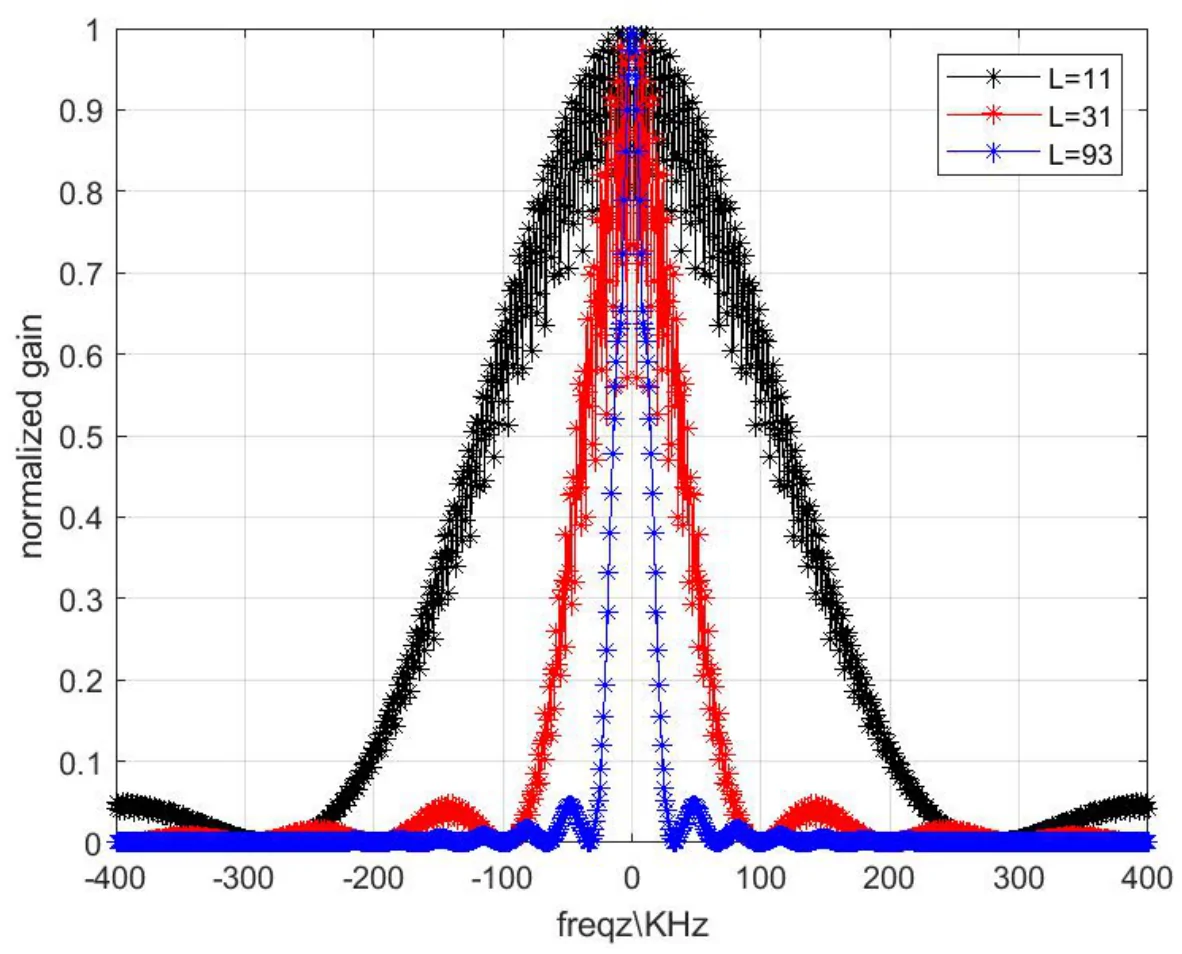
Impact of differently matched filter lengths on the PMF–FFT gain.

In order to reduce the number of parallel acquisition channels and minimize computational resource consumption, the length of the partial matched filter (PMF) L should generally not be set too long [19]. Figure 4 illustrates the normalized PMF–FFT gain obtained from the correlation of 100 sets of spreading codes at a code rate $R_{c}$ = $3.069\times 10^{6}$ chips/s. The horizontal axis represents the Doppler frequency offset, while the vertical axis shows the normalized magnitude of the gain.

It can be observed that when L = 93, the gain decreases rapidly with increasing Doppler offset. For example, at $f_{d}$ = 33 kHz, the gain has already decreased to zero, and the 3 dB bandwidth is only approximately 13 kHz. In contrast, when L = 11, the 3 dB gain bandwidth extends to nearly 100 kHz, indicating a significantly improved tolerance with the Doppler shift.

The hardware resource utilization (FFT modules) and the corresponding relationship with the choices of L and K are shown in the Table 2 below. The number of parallel acquisition branches required is estimated based on Figure 4, specifically when the PMF–FFT gain drops to 0.7071. When L = 11, the Doppler tolerance is approximately ±70 kHz; when L = 31, it is ±30 kHz; and when L = 93, it is ±10 kHz. Therefore, to cover a Doppler range of ±350 kHz, approximately 5, 12, and 35 parallel branches are needed, respectively.

### 3.4. Estimation of Acquisition Time

The coherent integration time is set to 40 ms. Assuming an 80 MHz system clock and the use of a radix-4 16,384-point FFT IP core, the estimated computational delay is approximately 0.77 ms. The postprocessing time required to analyze the PMF–FFT output is approximately $16,384\times T_{c}\approx$ 0.2 ms, where $T_{c}$ is the duration time of one chip. Therefore, the total processing time for a single coherent accumulation cycle is approximately 41 ms.

Given a total of 1023 possible code phase positions and assuming a uniform random distribution of the initial phase, the expected time to acquire the signal in a single full scan is approximately 20.97 s. To reduce acquisition time, a dual-search strategy can be implemented: one local correlator scans from code phase position 0, while another simultaneously scans from position 512. This parallel approach effectively reduces the expected acquisition time to approximately 10.48 s. However, the trade-off is that the number of parallel acquisition channels must be doubled from 5 to 10, thereby doubling the hardware resource consumption of the FFT computation modules.

### 3.5. PMF–FFT-Based Multi-Path Parallel High-Sensitivity Acquisition Algorithm for a Wide Doppler Range

The acquisition of DSSS–GMSK relies on detecting the pilot signal by generating a locally produced code aligned in phase with the pilot. Once the pilot branch is acquired, the Doppler-consistent phase relationship between the pilot and signal branches is exploited to generate the local spreading code and carrier for the signal branch, thereby completing acquisition.

The intermediate-frequency (IF) input signal, after DC filtering and digital automatic gain control (AGC), can be expressed as follows:

(9) $$\begin{array}{c} S_{rev}\left(t\right)=cos\left(2\pi f_{c}t+f_{d}t+\varphi_{1}+G_{0}\left(t\right)\right)+cos\left(2\pi f_{c}t+f_{d}t+\varphi_{1}+G_{1}\left(t\right)\right)+N_{noise} \end{array}$$

where $f_{d}$ denotes the Doppler frequency shift of the received signal; $G_{0}$ denotes the phase modulation of the navigation branch; $G_{1}$ denotes the phase modulation of the data branch; and $N_{noise}$ corresponds to the additive white noise introduced by the channel.

By applying the Laurent decomposition, the received DSSS–GMSK signal can be expanded into multiple components. The result can be interpreted as comprising two parts: the 0th-order component, which closely resembles the exact Laurent decomposition of MSK, and the remaining higher-order components. The Laurent decomposition can be expressed as follows:

(10) $$\begin{array}{c} e^{jG_{0}\left(t\right)}=\beta_{0}+\sum_{k=1}^{2^{L-1}-1}\sum_{n=0}^{\infty }c_{n,k}C_{k}\left(t-nT_{c}\right) \end{array}$$

When choosing BT = 0.3 and number of symbols L = 3, more than 99% signal energy is concentrated in the 0th-order component [15]. In the subsequent analysis, only the coupling among the local carrier, the spreading code, and the 0th-order component is considered. The 0th-order component can be approximated as follows:

(11) $$\begin{array}{c} \beta_{0}≃a_{i}\left(t\right)cos\left(\frac{\pi t}{2T_{c}}\right)+ja_{q}\left(t-T_{c}\right)sin\left(\frac{\pi t}{2T_{c}}\right) \end{array}$$

As a result, Equation (9) can be approximately expressed as follows:

(12) $$\begin{array}{c} S_{rev}\left(t\right)≃a_{i}\left(t\right)cos\left(\frac{\pi t}{2T_{c}}+\varphi_{0}\right)cos\left(2\pi f_{c}t+f_{d}t+\varphi_{1}\right)+a_{q}\left(t-T_{c}\right)sin\left(\frac{\pi t}{2T_{c}}+\varphi_{0}\right)sin\left(2\pi f_{c}t+f_{d}t+\varphi_{1}\right)+\\ b_{i}\left(t\right)cos\left(\frac{\pi t}{2T_{c}}+\varphi_{0}\right)cos\left(2\pi f_{c}t+f_{d}t+\varphi_{1}\right)+b_{q}\left(t-T_{c}\right)sin\left(\frac{\pi t}{2T_{c}}+\varphi_{0}\right)sin\left(2\pi f_{c}t+f_{d}t+\varphi_{1}\right)+N_{noise} \end{array}$$

$a_{i}\left(t\right)$ represents the pulse-shaping sequence corresponding to the even-indexed differentially encoded symbols of pilot code $c_{n}$, while $a_{q}\left(t\right)$ corresponds to the odd-indexed differentially encoded symbols of pilot code $c_{n}$, with both sequences having a period of approximately 2$T_{c}$. Accordingly, $b_{i}\left(t\right)$ represents the pulse-shaping sequence for the even bits (differentially encoded $b_{n}$), while $b_{q}\left(t\right)$ corresponds to that for the odd bits.

$S_{rev}\left(t\right)$ is multiplied by locally generated carriers $\cos\left(2\pi f_{i}t\right)\cos\left(R_{c}t/2\right)$, $-\sin\left(2\pi f_{i}t\right)\sin\left(R_{c}t/2\right)$,$-\sin\left(2\pi f_{i}t\right)\cos\left(R_{c}t/2\right)$ and $\cos\left(2\pi f_{i}t\right)\sin\left(R_{c}t/2\right)$ with distinct center frequencies, where i = 0, 1, 2, 3, 4 corresponds to the five different local center frequencies [15]. After passing through a low-pass filter with bandwidth $R_{c}/2$ (a high-order CIC filter is adopted in this paper), the results can be expressed as follows:

(13) $$S_{1}=\frac{1}{4}a_{i}\left(t\right)cos\left(2\pi \left(f_{c}-f_{i}\right)t+f_{d}t+\varphi_{1}\right)cos\left(\varphi_{0}\right)+\frac{1}{4}a_{q}\left(t-t_{c}\right)sin\left(2\pi \left(f_{c}-f_{i}\right)t+f_{d}t+\varphi_{1}\right)sin\left(\varphi_{0}\right)+N_{noise}^{1}$$

(14) $$S_{2}=-\frac{1}{4}a_{i}\left(t\right)sin\left(2\pi \left(f_{c}-f_{i}\right)t+f_{d}t+\varphi_{1}\right)sin\left(\varphi_{0}\right)-\frac{1}{4}a_{q}\left(t-t_{c}\right)cos\left(2\pi \left(f_{c}-f_{i}\right)t+f_{d}t+\varphi_{1}\right)cos\left(\varphi_{0}\right)+N_{noise}^{2}$$

(15) $$S_{3}=\frac{1}{4}a_{i}\left(t\right)sin\left(2\pi \left(f_{c}-f_{i}\right)t+f_{d}t+\varphi_{1}\right)cos\left(\varphi_{0}\right)-\frac{1}{4}a_{q}\left(t-t_{c}\right)cos\left(2\pi \left(f_{c}-f_{i}\right)t+f_{d}t+\varphi_{1}\right)sin\left(\varphi_{0}\right)+N_{noise}^{3}$$

(16) $$S_{4}=-\frac{1}{4}a_{i}\left(t\right)cos\left(2\pi \left(f_{c}-f_{i}\right)t+f_{d}t+\varphi_{1}\right)sin\left(\varphi_{0}\right)+\frac{1}{4}a_{q}\left(t-t_{c}\right)sin\left(2\pi \left(f_{c}-f_{i}\right)t+f_{d}t+\varphi_{1}\right)cos\left(\varphi_{0}\right)+N_{noise}^{4}$$

Define $I=S_{1}+S_{2}+\left(S_{3}-S_{4}\right)j$ and $Q=-S_{2}-S_{1}+\left(S_{4}-S_{3}\right)j$. I branch and Q branch can be expressed as follows:

(17) $$I\left(t\right)=\frac{1}{4}a_{i}\left(t\right)e^{j(2\pi \left(f_{c}-f_{i}\right)t+f_{d}t+\varphi_{1}+\varphi_{0})}-\frac{1}{4}a_{q}\left(t-t_{c}\right)je^{j(2\pi \left(f_{c}-f_{i}\right)t+f_{d}t+\varphi_{1}+\varphi_{0})}+N_{noise}^{1}+N_{noise}^{2}+1j\left(N_{noise}^{3}-N_{noise}^{4}\right)$$

(18) $$Q\left(t\right)=-\frac{1}{4}a_{i}\left(t\right)e^{j(2\pi \left(f_{c}-f_{i}\right)t+f_{d}t+\varphi_{1}+\varphi_{0})}+\frac{1}{4}a_{q}\left(t-t_{c}\right)je^{j(2\pi \left(f_{c}-f_{i}\right)t+f_{d}t+\varphi_{1}+\varphi_{0})}-N_{noise}^{1}+N_{noise}^{2}-1j\left(N_{noise}^{3}-N_{noise}^{4}\right)$$

Both branches are downsampled at intervals corresponding to different Doppler shifts, with the I branch delayed by T relative to Q. The resulting $I_{ud}$ and $Q_{ud}$ are deinterleaved into $C_{rev}$ and fed into the PMF–FFT module [19]. Let $\phi \left(t\right)=2\pi \left(f_{c}-f_{i}\right)t+f_{d}t+\varphi_{1}+\varphi_{0}$ and the $C_{rev}$ can be expressed as follows:

(19) $$\;C_{rev}=\frac{1}{4}a_{i}\left(0\right)-\frac{1}{4}a_{q}\left(-t_{c}\right),\frac{1}{4}a_{q}\left(0\right)e^{j\phi (t_{c})}-\frac{1}{4}a_{i}\left(t_{c}\right)e^{j\phi (t_{c})},\frac{1}{4}a_{i}\left(2t_{c}\right)e^{j\phi (2t_{c})}-\frac{1}{4}a_{q}\left(t_{c}\right)e^{j\phi (2t_{c})},...$$

**Figure 5:**
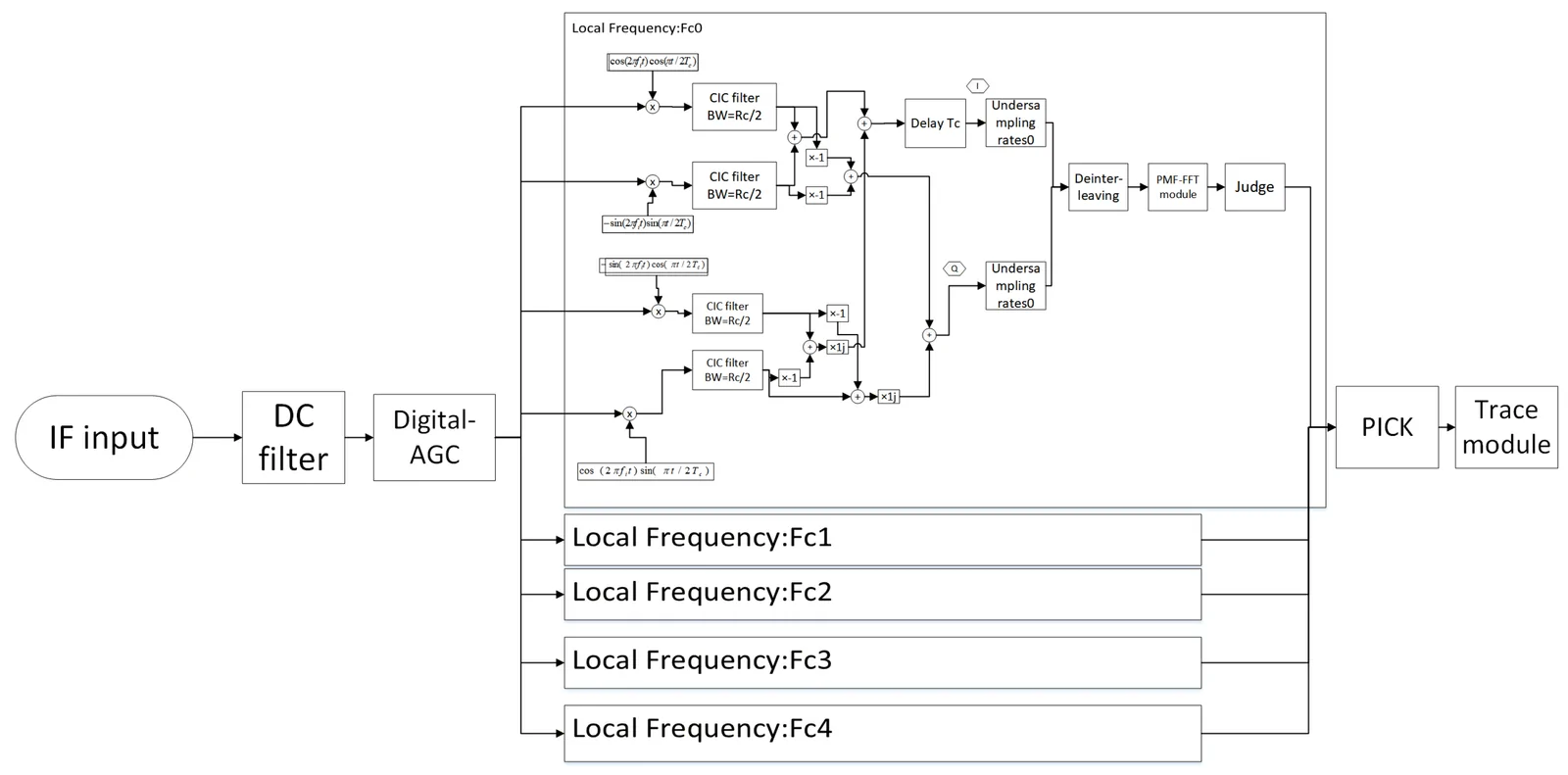
DSSS–GMSK signal acquisition algorithm.

The overall processing flow is illustrated in Figure 5.

After deinterleaving, the signal $C_{rev}$ is subjected to partial correlation with the locally generated pseudo-noise (PN) code (differentially encoded) over L points. This operation is repeated K times to cover different code-phase offsets. The resulting K correlation values are stored in block RAM (BRAM). Subsequently, these values are forwarded to a Fast Fourier-Transform (FFT) module operating at a higher clock frequency. In order to match the input length required by the FFT and to improve frequency resolution, zero padding is applied to extend the data from length K to N (where N is typically chosen as a power of two for implementation efficiency on FPGA platforms). This zero padding also helps alleviate the scalloping loss inherent in FFT processing [19].

From the N-point FFT output, the following spectral characteristics are extracted:

$P_{max0}^{i}$: the maximum magnitude (that is, the dominant spectral peak)’

$P_{max1}^{i}$: the second-highest magnitude’

$P_{avg}^{i}$: the average magnitude across all frequency bins.

A signal is successfully acquired if the following conditions are simultaneously satisfied:

(20) $$\begin{array}{c} \left|\frac{P_{max0}^{i}}{P_{avg}^{i}}\right|>22,\left|\frac{P_{max1}^{i}}{P_{max0}^{i}}\right|>1.2 \end{array}$$

**Figure 6:**
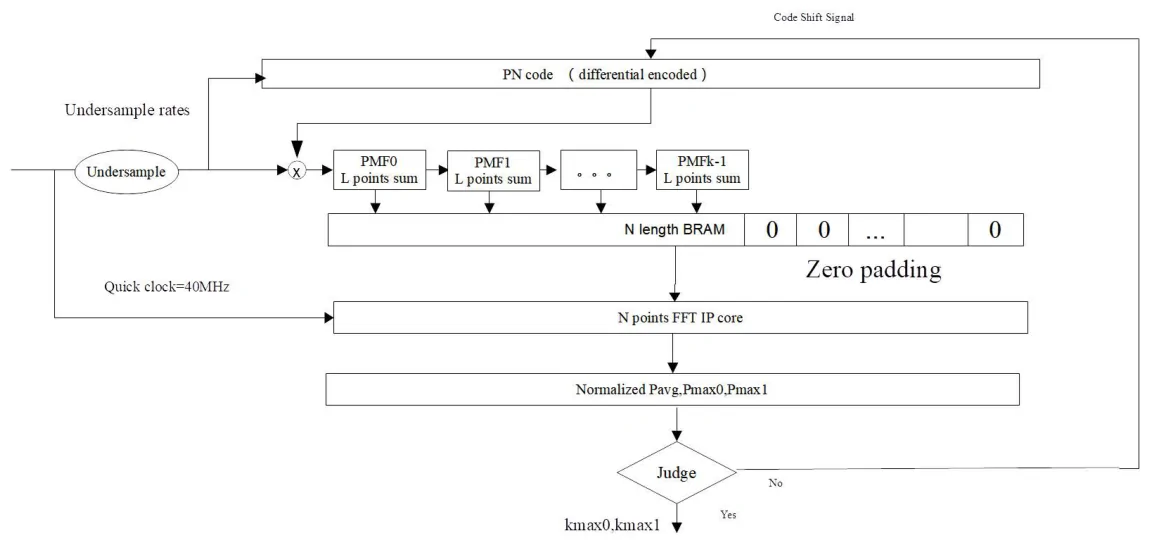
Logic of PMF–FFT module.

If these conditions are not met, the local differentially encoded PN code is shifted by one-chip phase, and the correlation and PMF–FFT calculations are repeated. The logic of the PMF–FFT module is illustrated in Figure 6. To enhance acquisition efficiency, multiple correlation branches can be processed in parallel. If multiple branches satisfy the acquisition criteria, the one with the largest $\left|\frac{P_{max0}^{i}}{P_{avg}^{i}}\right|$ is selected as the reference for subsequent Doppler frequency estimation.

The rationale behind setting the primary-to-average magnitude ratio threshold above 22 is to increase acquisition sensitivity and robustness. Additionally, constraining the ratio between the primary and secondary peaks helps to reduce the false acquisition alarm near the detection threshold.

## 4. Simulation Conditions and Results

### 4.1. Simulation Results of PMF–FFT-Based Multi-Path Parallel Acquisition Algorithm

The pre-simulation tool is implemented using MATLAB R2024b.The channel model adopts the CDL-E channel model, in which some of the capture modules have already been implemented in Verilog. The modulation scheme is DSSS–GMSK, with a Gaussian filter bandwidth-time product BT=0.3, and the number of symbols L is 3. The receiving carrier frequency is $f_{1}$ = 7.23 GHz, and the PN code rate is $R_{c}$ = $3.069\times 10^{6}$ chips/s. The Doppler acquisition range is set to $f_{d}$ = −350 kHz∼350 kHz with a Doppler changing rate of 3 kHz/s. In this work, we consider the Earth–Moon satellite communication scenario. Since multipath propagation can be considered nearly negligible, the channel is modeled as a large-scale fading channel with a Rician K-factor of 22 dB [20]. The channel coefficient h is generated as follows:

(21) $$\begin{array}{c} h=\sqrt{\frac{K}{K+1}}e^{j\theta }+\sqrt{\frac{1}{K+1}}z \end{array}$$

θ∼U[0,2π] and z∼CN(0,1) is a standard complex Gaussian random variable representing the aggregate scattered components from all non-LOS (NLOS) clusters. The sampling rate is set as 40M sample/s. After down-conversion, the intermediate frequency (IF) center frequency is $f_{c}=4\;MHz$. The partial matched filter length L is 11. 119 complete code periods are collected, leading to K = 93×119 and an integration duration of 39.7 ms. The FFT size N satisfies N > K, with N = 16,384 selected for computation.

Five local carriers are generated with center frequencies of $f_{c}-280$ kHz, $f_{c}-140$ kHz, $f_{c}$,$f_{c}+140$ kHz, and $f_{c}+280$ kHz, respectively. The corresponding downsampling rates can be calculated by Equation (23), which are 3,068,881 samples/s, 3,068,940 samples/s, 3,069,000 samples/s, 3,069,059 samples/s, and 3,069,119 samples/s.

(22) $$\begin{array}{c} R_{s}=R_{c}\left(1+\frac{dopplor}{f_{1}}\right) \end{array}$$

**Figure 7:**
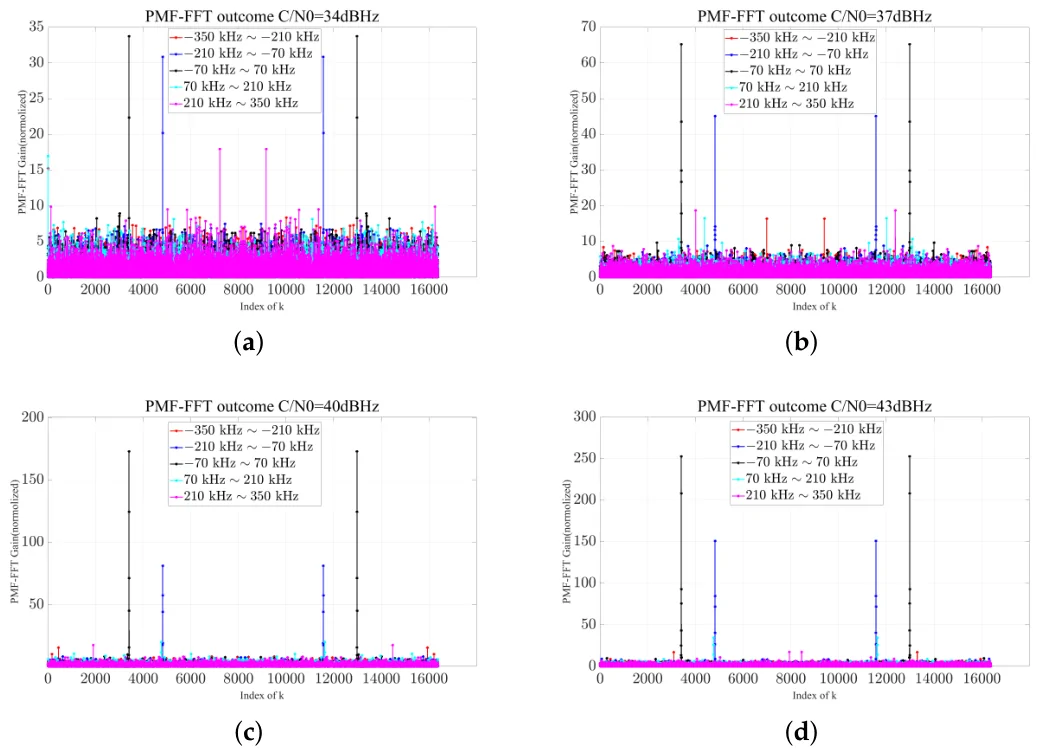
PMF–FFT module outputs for five branches when the received spread-spectrum code phase is aligned with the locally generated code phase. The horizontal axis represents the output sequence index of the PMF–FFT module (ranging from 1 to 16,384), and the vertical axis represents the magnitude of the correlation peak. (**a**) $C/N_{0}$ = 34 dB-Hz. (**b**) $C/N_{0}$ = 37 dB-Hz. (**c**) $C/N_{0}$ = 40 dB-Hz. (**d**) $C/N_{0}$ = 43 dB-Hz.

Figure 7 presents the simulation results. The horizontal axis represents the PMF–FFT output sequence index from 1 to 16,384, and the vertical axis indicates the magnitude of the correlation peak, expressed as $\left|\frac{P_{max0}^{i}}{P_{avg}^{i}}\right|$. The four subplots (top left, top right, bottom left, bottom right) correspond to $C/N_{0}$ values of 34 dB-Hz, 37 dB-Hz, 40 dB-Hz, and 43 dB-Hz, respectively, equivalent to received signal power levels from −138 dBm to −129 dBm. In each subplot, the five curves represent the results from five parallel acquisition branches with different down-conversion and down-sampling rates settings. The initial Doppler is set at −58 kHz, with a Doppler rate of +3 kHz/s. The results correspond to the case where the local code phase and the received signal code phase are aligned. It can be observed that the normalized correlation peak remains significant and exceeds the threshold of 22 even at a low $C/N_{0}$ of 34 dB-Hz. Among the five branches, the third branch (black line, center frequency at 0 kHz) shows the highest peak, with notable peaks at indexes 3405 and 12,981. The second branch (blue line, center frequency at −140 kHz) shows the second-highest peak, at indices 4819 and 11,567. Therefore, the Doppler frequency is determined to lie within the range of −140 kHz to 0 kHz.

(23) $$\begin{array}{ccc} f_{d} & = & f_{c}^{i}+\left(\frac{n}{L}+\frac{k}{NL}\right)R_{c}^{i} \end{array}$$

In Equation (23), $f_{c}^{i}$ is the center frequency, N = 16,384, L = 11; $R_{c}^{i}$ is the down-sampling rate; k is the FFT peak index; and n is an integer. Initially, the analysis focuses on the largest branch $\left|\frac{P_{max0}^{i}}{P_{avg}^{i}}\right|$ (as illustrated in the figure). When k = 3405, the possible values of $f_{d}$ are calculated by Equation (23) as −221.017 kHz corresponding to n=−1 and 57.983 kHz corresponding to n=0. When k = 12,981, the possible $f_{d}$ values are −57.949 kHz for n=1 and 221.050 kHz for n=0. Restricting the analysis to values within the range between the primary and secondary maxima (i.e., −140 kHz to 0 kHz), there exists a unique solution at k=12,981 and n=1, which produces a calculated $f_{d}$ of −57.949 kHz.

Subsequently, considering the second largest branch as a reference and again focusing on values within the 140 to 0 kHz interval, a possible solution is identified at k = 4819 with n = 0, resulting in a calculated $f_{d}$ of 57.938 kHz. This result closely matches the one obtained from the third-order branch.

**Figure 8:**
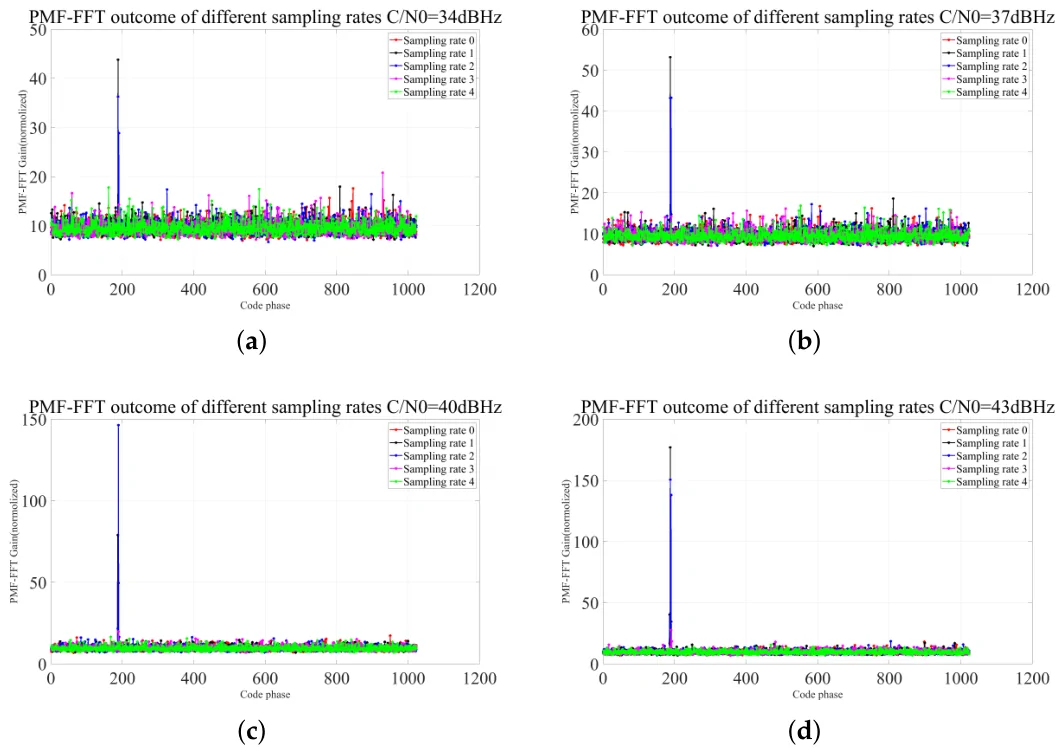
The results of coherent integration at different code phases for a five-channel. (**a**) $C/N_{0}$ = 34 dB-Hz. (**b**) $C/N_{0}$ = 37 dB-Hz. (**c**) $C/N_{0}$ = 40 dB-Hz. (**d**) $C/N_{0}$ = 43 dB-Hz.

Figure 8 illustrates the results of the local code in different code phases. The horizontal axis represents the chip offset, ranging from 1 to 1023, corresponding to the 1023 possible code positions. The vertical axis denotes the normalized coherent gain. Different colored lines represent the coherent gain results from five channels. The five sampling rates are 3,068,881 samples/s, 3,068,940 samples/s, 3,069,000 samples/s, 3,069,059 samples/s, and 3,069,119 samples/s, respectively. All coherent gain values have been normalized. It can be seen that once the code phase aligns, a distinct correlation peak appears at the $C/N_{0}$ threshold. A prominent coherent peak occurs at the simulated code phase of 200 (indicated by the black and blue lines). However, when the downsampling period does not match the code drift effect, the coherent gain is significantly reduced even if the local code phase is aligned and $C/N_{0}$ is relatively strong (as shown by the red, green, and purple lines). In long-term integration, code drift will cause invalid coherent accumulation and introduce additional noise.

### 4.2. Performance Against False Locking Between Different Code Groups Using the Same Frequency Point

In a co-frequency environment, multiple code groups can be modulated at the same frequency point, where these codes have the same chip rate and period as the target code intended for acquisition. To achieve higher sensitivity, a lower detection threshold is often set, which increases the risk of false locking due to coherent accumulation with other codes present at the same frequency. Therefore, an optimized decision method is required to mitigate the risk of false acquisition. In this study, multiple groups of random codes were generated for simulation. Each random code had the same power as the target code intended for acquisition. After superimposing the random codes onto the target spread-spectrum signal, noise was added corresponding to a carrier-to-noise density ratio $C/N_{0}$ of 34 dB-Hz. Parallel acquisition calculations were performed using the proposed method, and the primary peak value, the average value, and the secondary peak value were collected. A total of 28,000 groups of random codes were generated, with random chip phases and Doppler shifts uniformly distributed within the range of −350 kHz to 350 kHz.

**Figure 9:**
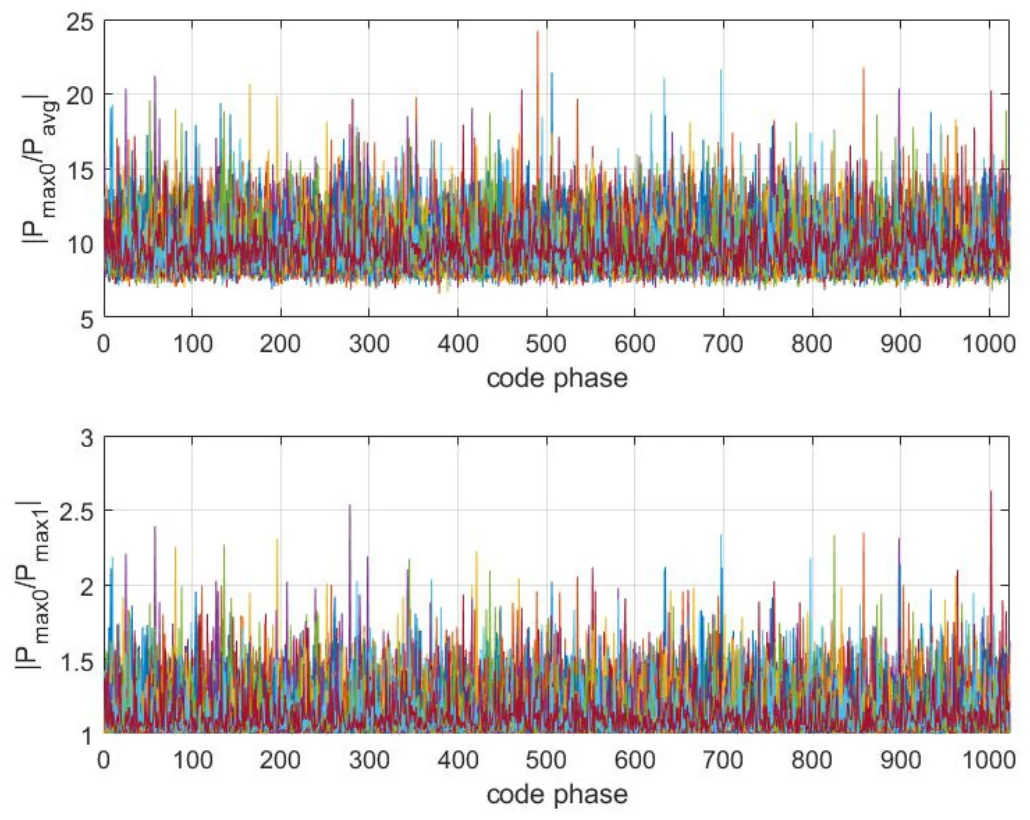
Coherent gain between same-frequency different code groups (left: maximum normalized gain; right: ratio of the maximum normalized gain to the secondary maximum normalized gain.

The results are shown in Figure 9. In the left panel, the horizontal axis represents the initial Doppler shift of the random codes, while the vertical axis shows the normalized primary peak value $\left|\frac{P_{max0}^{i}}{P_{avg}^{i}}\right|$ obtained after PMF–FFT processing. It can be seen that among the 28,000 cases, only one normalized result exceeded the threshold of 22, with a value of 24.22. In the right panel, the vertical axis represents the ratio of the primary peak to the secondary peak $\left|\frac{P_{max0}^{i}}{P_{max1}^{i}}\right|$ after PMF–FFT normalization. When the maximum value of 24.22 occurred, the primary-to-secondary peak ratio was 1.72, which does not satisfy the false alarm condition (the primary-to-secondary ratio must be less than 1.2 to indicate a false alarm). By supplementing primary peak detection with an additional criterion based on the primary-to-secondary peak ratio, false acquisitions caused by noise and interference from other random codes can be effectively suppressed.

## 5. Conclusions

This paper introduces a Direct-Sequence Spread-Spectrum Gaussian-filtered Minimum-Shift Keying (DSSS–GMSK) modulation scheme suitable for Deep-Space Telemetry, Tracking, and Control communications, and explains its advantages over the commonly employed DSSS–BPSK approach. To demonstrate its feasibility, the paper further analyzes the acquisition problem for DSSS–GMSK signals under high-sensitivity and wide Doppler dynamic range conditions. The integration time for successful acquisition is estimated, and a parallel acquisition algorithm based on the PMF–FFT method is proposed. The approach enables the acquisition of DSSS–GMSK signals with Doppler shifts up to ±350 kHz at a carrier-to-noise density ratio $\left(C/N_{0}\right)$ of 34 dB-Hz (SNR = −33.8 dB). The proposed system performs ten 16,384-point FFT operations in parallel with a 40 MHz clock, achieving an estimated acquisition time on the order of 10 s. This method mitigates code-phase drift caused by long coherent integration periods and employs a detection strategy based on the primary peak, secondary peak, and mean value to ensure high sensitivity while minimizing the risk of false acquisition alarms. The trade-offs between acquisition time and computational hardware resources are analyzed, and the feasibility of the method is verified through simulation.

Future work will focus on refining the research on the tracking and demodulation module for DSSS–GMSK and conducting engineering studies for on-board satellite integration. We plan to investigate the application of JPL PN codes to DSSS–GMSK signals. This approach is expected to enhance the anti-interference performance of the spreading code and improve ranging accuracy in low-SNR environments. The feasibility of the complete engineering modification will be thoroughly discussed. The DSSS–GMSK scheme will be promoted as a pilot implementation in several Earth–Moon space science exploration satellite projects at the Chinese Academy of Sciences, followed by in-orbit verification. The ultimate goal is to establish a relatively standardized specification, positioning it as an alternative spread-spectrum solution for Earth–Moon deep-space applications.

## Figures and Tables

**Table 1:** Uplink calculation for the Earth–Moon transfer orbit.

Item	Unit	Value
Uplink Frequency	MHz	7200
EIRP	dBW	78
Distance	km	1,500,000
Free-Space Loss	dB	233.11
Atmospheric Loss	dB	1
Antenna Pointing Loss	dB	1.5
Polarization Loss	dB	1
Receiver Channel Insertion Loss	dB	3
System Receiving G/T(Omnidirectional Reception)	dB/K	−32
Received Power at the Transponder Inputs	dBm	−138.38
Receiver Noise Figure	dB	2.5
$C/N_{0}$	dB-Hz	34

**Table 2:** Resource consumption of FFT modules corresponding to different combinations.

Modulation	L (Partial Correlation Points)	K (Number of Correlation)	N (FFT Lengths)	Resources of Single FFT Channel	Number of Parallel Acquisition Channels (Cover −350∼350 kHz)	Total Resources
DSSS–GMSK	11	11160	16,384	DSP48 slice 44 Brams	5	45 DSP48 slice + 220 Brams
DSSS–GMSK	31	3960	4096	DSP48 slice 44 Brams	12	45 DSP48 slice + 220 Brams
DSSS–GMSK	93	1320	2048	DSP48 slice 44 Brams	35	45 DSP48 slice + 220 Brams

## Data Availability

No new data were created or analyzed in this study. Data sharing is not applicable to this article.
